# Comparison of nutritional risk index and body mass index in predicting survival outcomes in patients undergoing allogeneic hematopoietic stem cell transplantation: a retrospective study

**DOI:** 10.1016/j.htct.2026.106254

**Published:** 2026-02-13

**Authors:** Bruna Silvestre Françoso, Isabela Laurêncio Schiavoni, Thauany Nantes Guirao, Barbara David dos Santos, Thalita Cristina de Mello Costa, Anderson Marliere Navarro, Fabiola Traina, Juliana Maria Faccioli Sicchieri

**Affiliations:** a Department of Internal Medicine, Ribeirão Preto Medical School, University of São Paulo, Brazil; b Department of Medical Imaging, Hematology and Clinical Oncology, Clinical Hospital of Ribeirão Preto Medical School, University of São Paulo, Brazil; c Department of Health Sciences, School of Medicine of Ribeirão Preto, University of São Paulo, Brazil

**Keywords:** Nutritional risk index (NRI), Allogeneic hematopoietic stem cell transplantation, Nutritional assessment, Nutritional screening, Malnutrition, Body mass index

## Abstract

**Introduction:**

Allogeneic hematopoietic stem cell transplantation is a viable therapeutic option for several serious diseases however it is a high-risk procedure because it involves high-toxicity protocols with many adverse effects. Existing factors, such as the underlying disease and nutritional status, may influence the outcome. The objective of this study was to evaluate the Nutritional Risk Index as a prognostic tool by correlating it with body mass index, nutritional status, and clinical outcomes in patients undergoing hematopoietic stem cell transplantation.

**Methods:**

This single center retrospective study was conducted collected sociodemographic, anthropometric, biochemical, and clinical data before conditioning and 30 days post-transplantation. Statistical analyses were performed using the Mann-Whitney test and Spearman's correlation. Overall survival was estimated using the Kaplan-Meier method, with comparisons conducted via the Gehan-Breslow-Wilcoxon test. A Cox Proportional Hazards regression analysis was employed to identify factors associated with mortality; variables demonstrating a p-value ≤0.20 in the univariate analysis were included in the multivariate model. For all analyses, statistical significance was defined as a p-value <0.05.

**Results:**

Seventy-seven participants were included, with an average age of 41 years. According to the nutritional risk index, the entire sample was classified as having severe nutritional risk. The body mass index showed that 6.4 % were malnourished, 19.4 % were obese, and 12.9 % had hypoalbuminemia. The estimated survival curve identified a significant difference for patients aged <45 years with survival being significantly longer (p-value = 0.01). Higher albumin levels (≥3.5) after transplantation were associated with longer survival (p-value = 0.04). Sex, body mass index, albumin level before conditioning, and graft-versus-host disease showed no significant differences in terms of survival. Albumin levels ≥3.5 g/dL after transplantation were marginally associated with a lower mortality risk and malignant disease showed a trend toward increased mortality.

**Conclusion:**

These findings underscore the clinical utility of prognostic indices, such as the Nutritional Risk Index and albumin levels, during the pre-transplant period, emphasizing the necessity for early nutritional interventions in hematopoietic stem cell transplantation patients.

## Introduction

Hematopoietic stem cell transplantation (HSCT) is a curative therapeutic option for several onco-hematological diseases [1]. In recent years, an increase in this procedure has been observed even with the COVID-19 pandemic: 3391 HSCT performed in Brazil in 2022 and 1026 in the first quarter of 2023 [2].

Although HSCT represents a viable therapeutic option for some serious diseases, it is a high-risk procedure, as it involves high-toxicity protocols, with many adverse effects such as mucositis, febrile neutropenia, and infection [1]. Some existing factors, such as the underlying disease, number of associated comorbidities, and nutritional status, may influence the treatment outcome; others directly related to HSCT, such as the intensity of the conditioning regimen and the occurrence of graft-versus-host disease (GvHD), have important nutritional repercussions such as malabsorptive conditions and deficiencies, malnutrition and obesity especially during corticosteroid therapy, changes in metabolic energy, such as an increase or decrease in basal energy expenditure, and various biochemical changes [1,3]. However, the nutritional assessment of patients before transplantation is not an easy task [4]. Often, weight gain due to drugs such as glucocorticoids results in camouflaging changes in metabolism and tissue loss, especially muscle mass [5].

The use of the Nutritional Risk Index (NRI) is expanding because of its ease of use and good correspondence as a predictor of the interference of nutritional status on the patient's clinical outcome in different clinical situations [6]. NRI is a low-cost instrument as it uses data from biochemical tests that are routinely used in transplant centers and easy-to-obtain measurements, such as weight [7].

There is still no consensus in the literature regarding the optimal nutritional assessment method for this population. However, some studies indicate that due to treatment characteristics (such as glucocorticoid use and high-dose chemotherapy) and disease factors (including increased basal metabolic rate and hyporexia), weight fluctuations can mask the true deterioration of a patient’s nutritional status. The objective of this study was to correlate NRI classification data with nutritional status, and clinical outcomes of patients undergoing HSCT [1].

## Methods

This retrospective, descriptive, and exploratory study analyzed data from the electronic medical records of patients who underwent HSCT from January 2015 to December 2019 in a single center in Brazil. The study was approved by the institution’s research ethics committee under the number 3.819.250.

The cohort included over 18-year-old individuals of both sexes diagnosed with neoplastic and non-neoplastic hematological diseases. The exclusion criteria were age under 18 years old, those receiving autologous transplantations and those who underwent more than one transplant.

Sociodemographic, anthropometric, biochemical, and clinical data were collected for the study population. The time points evaluated were at the beginning of conditioning (T0) and 30 days post-transplantation (T1). The following formula was used to calculate the NRI [7]:

NRI=(1.519×serumalbumin(g/L)+41.7×(presentweight/usualweight)

The following classifications were used to stratify the risk of malnutrition: no nutritional risk (NRI ≥100), mild risk (97.5 ≤ NRI < 100), moderate risk (83.5 ≤ NRI < 97.5), and severe risk (NRI <83.5). Considering data from previous studies, it was assumed that NRI <97.5 would indicate clinically significant malnutrition [6,8,9]. In this study, patients did not receive standardized nutritional support protocols. Parenteral nutrition was initiated only in specific clinical situations, particularly in cases of severe hyporexia and Grade 4 mucositis in which oral intake is not feasible. None of the patients received enteral nutrition during the study period. Therefore, nutritional management was individualized based on clinical judgment and patient tolerance. The primary outcomes were the NRI classifications before and after HSCT. These classifications were analyzed in relation to sex, etiopathogenesis (neoplastic versus non-neoplastic), and clinical outcomes, including GvHD, disease recurrence, and mortality.

Statistical analyses were performed using the STATA software employing the Mann-Whitney non-parametric test for numerical variables (NRI, albumin, and body mass index [BMI]). The non-parametric Kruskal-Wallis test was used to analyze the albumin levels at different time points. For NRI and BMI, the Wilcoxon signed-rank test was applied to paired samples. The correlation between NRI (before and after) and albumin level (before and after) was assessed using Spearman's rank correlation coefficient.

Overall survival curves were estimated using the Kaplan-Meier method and compared between groups using the Gehan-Breslow-Wilcoxon test, considering variables such as age, sex, BMI, albumin levels, and disease type. In addition, Cox proportional hazards regression was performed by including variables with p-values ≤0.20 in the univariate analysis. Statistical significance was defined as p-value <0.05 for all analyses.

## Results

Table 1 summarizes the clinical characteristics of the study population. During the study period, a total of 151 transplants were performed at the center. After the exclusion of 48 pediatric patients and 41 patients who underwent a second transplant or autologous HSCT, the final cohort comprised 77 participants with a mean age of 41 years. Hematological neoplasms accounted for 77 % of the sample. Additionally, over 80 % of transplants were performed using related-donor grafts, with 67 % of stem cells being derived from bone marrow.

**Table 1:** Sample characterization.

		n	%
**Sex**	Female	34	44.2
	Male	43	55.8
**Illness**	Malignant	59	76.6
	Not Malignant	18	23.4
**BMI**	Normal	57	74
	Underweight	5	6.4
	Obese	15	19.4
**NRI**	Severe	77	100
	Moderate	0	0
	Mild	0	0
	No risk	0	0
**Albumin**	>3.5	67	87
	<3.5	10	12.9
**Progenitor cell source**	Bone Marrow	52	67.5
	Peripheral blood	24	31.2
	Umbilical cord	1	1.3
**Related Donor**	Related	62	80.5
	Unrelated	15	19.5
**Conditioning Regimen**	Myeloablative	50	64.9
	Non-myeloablative	5	6.5
	Reduced Intensity	22	28.6
**GvHD**	Yes	28	36.4
	No	49	63.6
**GvHD site**	Skin	10	13.0
	Mouth	2	2.6
	Gastrointestinal tract	2	2.6
	Liver	1	1.3
	≥2 sites	13	16.9
**Outcome**	Remission	55	71.4
	Others*	22	28.6

GvHD, graft-versus-host disease; BMI, body mass index; NRI, nutritional risk index;.

⁎ Note: Other outcomes included relapse and death.

Data on the conditioning regimen are presented In Table 1. About 65 % of the sample received the myeloablative protocol. Approximately 28 % of patients were diagnosed with GvHD, with the highest incidence (13 %) being skin GvHD. Approximately 71 % of the patients were in remission and 28.6 % died or relapsed.

The NRI values were significantly below the clinical threshold, classifying all patients as having a severe risk of malnutrition at baseline (T0; mean: 49.37). These values remained low at the subsequent assessment (T1; mean: 47.54), showing a significant decline (p-value = 0.003). Similarly, the BMI values differed significantly (p-value = 0.01) between T0 (mean: 25.44) and T1 (mean: 25.08). Despite this decrease, the participants remained classified as overweight at both time points (Table 2).

**Table 2:** Comparing nutritional risk indexes and body mass indexes at the beginning of conditioning (T0) and 30 days post-transplantation (T1).

Index	T0 (*n* = 77)	T1 (*n* = 77)	p-value
NRI	49.29 ± 7.57	47.54 ± 4.76	0.003
BMI (kg/m²)	25.44 ± 5.23	25.08 ± 4.89	0.01

NRI: Nutritional risk index; BMI: Body mass index.

The mean albumin values at T0 and T1 were 3.89 ± 0.39 and 3.88 ± 0.60, respectively. Table 3 shows the correlation between NRI and albumin levels. The NRI T1 score was positively correlated with the T1 albumin level (*r* = 0.485; p-value = 0.000).

**Table 3:** Correlation of nutritional risk index with albumin level.

	NRI T0 (*n* = 77)	NRI T1 (*n* = 77)
Albumin T0	*r* = 0.148	*r* = 0.35
	p-value = 0.199	p-value = 0.766
Albumin T1	*r* = 0.160	*r* = 0.485
	p-value = 0.166	p-value = 0.000

NRI: Nutritional Risk Index.

On comparing albumin levels with the outcome, significantly higher albumin values at T0 were found in the remission group (p-value = 0.048). The NRI score and BMI did not differ significantly between the outcomes (Table 4).

**Table 4:** Comparison of outcomes with albumin, nutritional risk index and body mass index.

	Remission (*n* = 55)	Other outcomes (*n* = 22)	p-value
Albumin T0 (g/dL)	3.96 ± 0.36	3.74 ± 0.44	0.048
Albumin T1 (g/dL)	3.97 ± 0.46	3.64 ± 0.83	0.102
NRI T0	49.92 ± 8.19	47.96 ± 4.92	0.420
NRI T1	49.98 ± 5.68	46.50 ± 4.25	0.470
BMI T0 (kg/m²)	25.59 ± 5.61	25.05 ± 4.25	0.866
BMI T1 (kg/m²)	25.16 ± 5.18	24.88 ± 4.20	0.752

NRI: Nutritional risk index; BMI: Body mass index.

*Note: Other outcomes were relapse and death.

When comparing albumin levels with the GvHD site, significantly higher albumin values at T0 were found in the two or More-site group (p-value = 0.056). The NRI score and BMI did not differ significantly between the GvHD site groups (Table 5).

**Table 5:** Comparison of graft-versus-host disease site with albumin, nutritional risk index and body mass index.

	Skin (*n* = 10)	Two or more sites (*n* = 13)	Other sites* (*n* = 5)	p-value
Albumin T0 (g/dL)	3.79 ± 0.30	3.89 ± 0.40	3.80 ± 0.53	0.056
Albumin T1 (g/dL)	3.62 ± 0.26	3.70 ± 0.37	4.00 ± 0.55	0.533
NRI T0	52.72 ± 13.19	46.94 ± 5.27	48.84 ± 3.71	0.592
NRI T1	47.06 ± 2.98	45.92 ± 2.40	47.59 ± 1.00	0.325
BMI T0 (kg/m²)	27.95 ± 6.96	23.29 ± 3.87	26.96 ± 7.61	0.284
BMI ‘(kg/m²)	26.55 ± 6.22	23.32 ± 3.73	26.56 ± 6.63	0.490

NRI: Nutritional risk index; BMI: Body mass index.

⁎ Note: Other sites included the mouth, gastrointestinal tract, and liver.

**Fig. 1:**
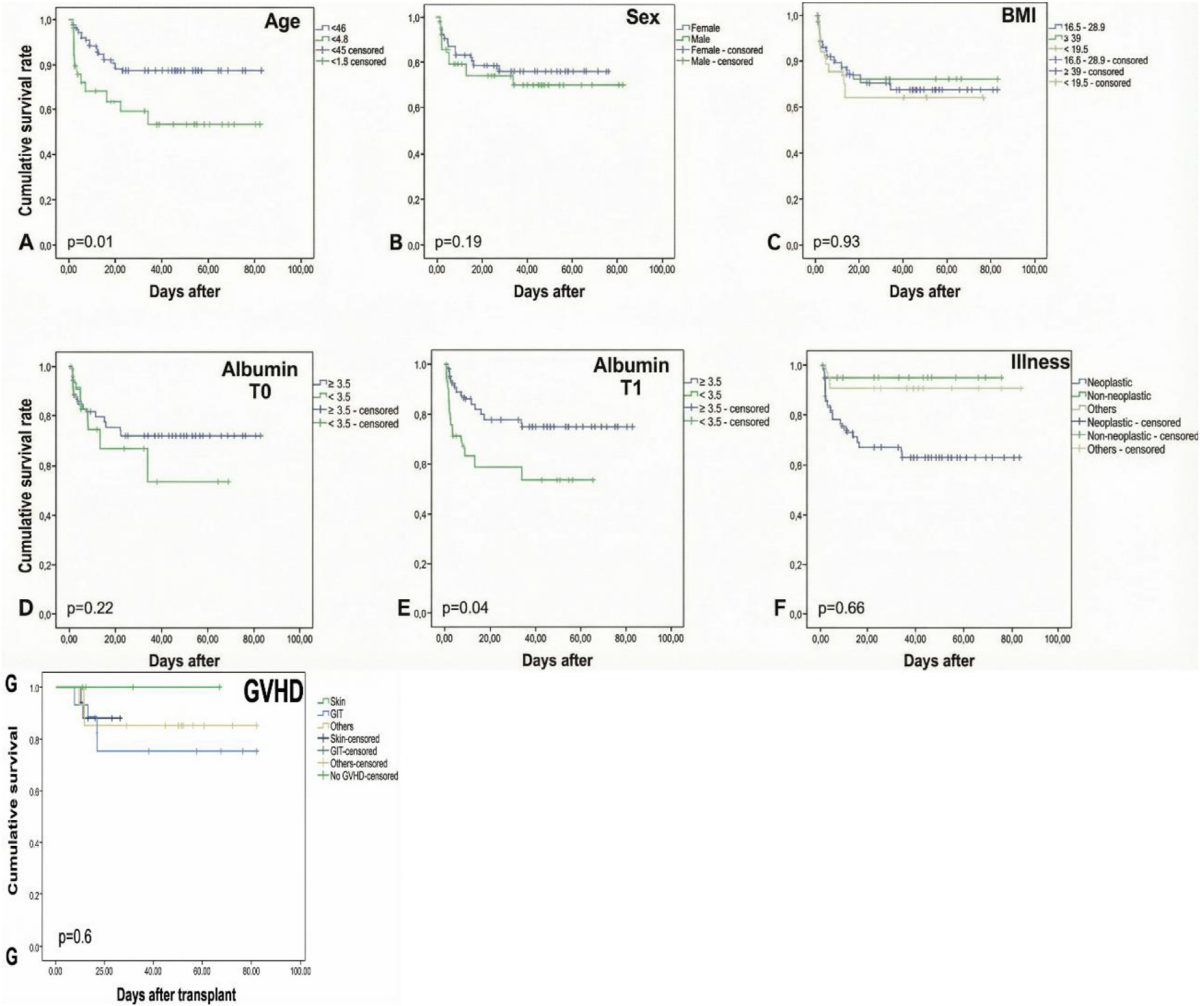
Survival curves.

The estimated survival curves (Figure 1) demonstrated that an age of <45 years was significantly associated with a higher survival rate (p-value = 0.01). Higher albumin levels (≥3.5 g/dL) at T1 were associated with longer survival (p-value = 0.04), whereas malignant diseases were associated with shorter survival (p-value = 0.006). Conversely, sex, body mass index (BMI), albumin levels at T0, and GvHD did not show any significant association with survival outcomes.

A: Estimated survival curve according to age. B: Estimated survival curve according to sex. C: Estimated survival curve based on body mass index. D: Estimated survival curve according to albumin level at T0. E: Estimated survival curve according to albumin level at T1. F: Estimated survival curve according to disease type. G: Estimated survival curve based on graft-versus-host disease

In the multivariate Cox regression analysis, sex (p-value = 0.36) and age (p-value = 0.42) were excluded from the model because of the lack of statistically significant associations with the outcome. Among the variables retained in the model, albumin levels ≥3.5 g/dL at time point T1 showed a marginal association with a lower risk of death (p-value = 0.07; OR = 0.40; 95 % CI: 0.15–1.07), suggesting a trend toward a 60 % reduction in the odds of mortality compared to lower levels. Similarly, the presence of malignant disease was associated with a trend toward increased risk of death (p-value = 0.06; OR = 7.03; 95 % CI: 0.89–55.44), corresponding to approximately sevenfold higher odds of mortality compared to other conditions, although this result was not statistically significant.

## Discussion

This study highlights the importance of using instruments for the early assessment of nutritional status combining biomarkers with anthropometric data, such as the NRI. Despite the sample exhibiting normal mean serum albumin levels and a mean BMI categorized as overweight prior to transplantation, severe nutritional risk was identified across the entire cohort. These findings reinforce the importance of selecting accurate predictive tools and the early initiation of nutritional monitoring.

Albumin and NRI data at T1 seem to be associated with difficulties in maintaining an adequate nutritional intake in this treatment period [1,10,11]. During conditioning, patients experience hyporexia, nausea, and mucositis, which limit satisfactory energy and protein intake [11]. Therefore, the positive and significant correlation in this period reflects difficulties related to nutritional support.

Regarding the comparison between NRI and BMI, the data showed a significant difference between time points for both indexes, but did not indicate differences in the respective classifications compared to the beginning of treatment. It is worth noting that comparing these data with those of Sagou et al., higher mean BMI and lower NRI values were observed, signaling different characteristics in the samples regarding nutritional status [7]. This finding may reflect population characteristics, such as a difference in the overweight and obese numbers in the Brazilian population, as well as a worsening of the nutritional quality of food associated with the consumption of ultra-processed foods [12].

Patients whose outcome was remission showed significantly higher serum albumin levels in the initial assessment when compared to the other outcome group (death & relapse). This finding may be associated with the mobilization of acute-phase response proteins during inflammation, indicating the clinical role of albumin as a biomarker [13].

With respect to survival, it is noteworthy that serum albumin at T1 was associated with greater survival, probably not only because it is a nutritional marker but also because it indicates inflammation. The post-transplant period is characterized by inflammation and, as such, there is a re-prioritization of the hepatic protein metabolism, which can result in reduced serum levels of negative acute-phase proteins, such as albumin.

The findings regarding albumin levels provided an important insight into potential prognostic factors in the transplant population, although the associations observed did not reach the conventional levels of statistical significance. The trend toward a protective effect of higher albumin levels (≥3.5 g/dL) on mortality aligns with previous studies that highlighted serum albumin as a surrogate marker of nutritional and inflammatory status, both of which are known to influence clinical outcomes in critically ill and immunocompromised patients [14,15]. Although the association was marginal (p-value = 0.07), the observed 60 % reduction in the odds of death suggests that albumin may play a clinically relevant role in risk stratification and should be further explored in larger, adequately-powered cohorts.

Similarly, the association between malignant disease and a seven-fold increase in the odds of mortality (p-value = 0.06) underscores the severity and complexity of outcomes in patients with underlying cancer, although this finding was not statistically significant. The wide confidence interval (0.89–55.44) reflects sample size limitations and heterogeneity but may indicate a meaningful trend. These results support the hypothesis that disease type, particularly malignancy, may significantly affect post-transplant prognosis, reinforcing the need for disease-specific approaches in clinical management and risk modeling.

This study has several limitations that should be considered when interpreting the findings. First, the potential for selection bias cannot be excluded, given that participants were included based on clinical and available nutritional data, which may have led to an underrepresentation of patients with more severe conditions. Second, the unicentric nature of the study may limit the generalizability of the results, as institutional protocols, patient profiles, previous nutritional conditions, and care practices may differ between centers. Additionally, the heterogeneity of the sample, which included both malignant and non-malignant conditions, may have introduced confounding effects owing to differences in disease trajectory, conditioning regimen intensity, and nutritional risk. Although these factors reflect the real-world complexity of clinical practice, they may have influenced the observed associations and should be addressed in future multicenter studies with more homogeneous populations and standardized protocols.

Despite this, the NRI appears to be an efficient instrument for screening patients who will undergo HSCT, allowing teams to organize themselves according to levels of nutritional care, ensure adequate nutritional intervention strategies, and contribute to better treatment outcomes.

## Conflicts of interest

The author declares no conflicts of interest.

## Data Availability

The data that support the findings of this study are available from the corresponding author upon reasonable request.
